# Amidoxime-functionalized bead cellulose for the decomposition of highly toxic organophosphates†

**DOI:** 10.1039/d1ra01125a

**Published:** 2021-05-18

**Authors:** Pavel Janoš, Oldřich Tokar, Marek Došek, Karel Mazanec, Petr Ryšánek, Martin Kormunda, Jiří Henych, Pavel Janoš

**Affiliations:** Faculty of the Environment, University of Jan Evangelista Purkyně Pasteurova 3632/16 400 96 Ústí nad Labem Czech Republic pavel.janos@ujep.cz; Iontosorb Comp. Anežky České 657 400 07 Ústí and Labem Czech Republic; Military Research Institute Veslařská 230 637 00 Brno Czech Republic; Faculty of Natural Sciences, University of Jan Evangelista Purkyně Pasteurova 3632/16 400 96 Ústí and Labem Czech Republic; Institute of Inorganic Chemistry, Czech Academy of Sciences 250 68 Hsinec-Řež Czech Republic

## Abstract

Regenerated bead cellulose is a promising material with excellent mechanical and rheological properties, ideally suited for advanced environmental applications. By introducing the amidoxime functional group into the glucose unit at the C-6 position, highly effective reactive sorbent was prepared and used to destroy priority hazardous substances such as organophosphate pesticides or nerve-paralytic chemical warfare agents (CWAs). Quantum mechanical (QM) calculations were performed to study the interactions of organophosphates with amidoxime functional groups at the molecular level. It was found that the energetic reaction barrier of the rate-limiting step is markedly reduced (from 31.40 to 11.37 kcal mol^−1^) in the case of the amidoxime-catalysed degradation of parathion methyl, which resulted in a dramatic increase in the degradation rate; this was fully confirmed by experiments, in which the pesticide degradation proceeded at the time scale of several hours (*t*_1/2_ = 20–30 hours at pH 7.22).

## Abstract 2

Amidoxime-functionalized bead cellulose accelerates the cleavage of phosphoester bonds in toxic organophosphates.

## Graphical abstract

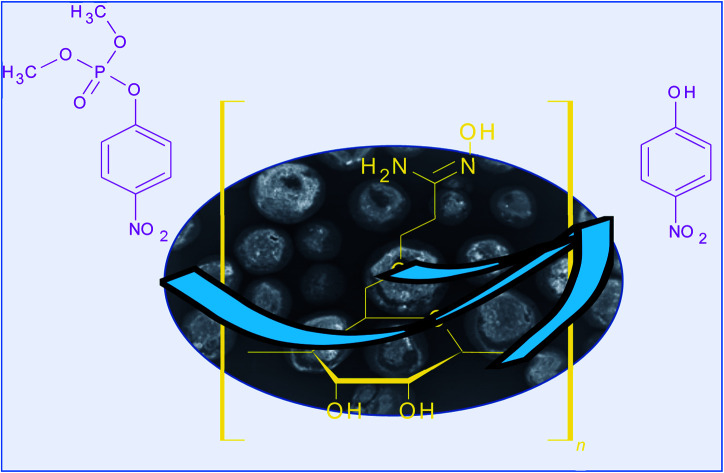

## 1 Introduction

The organophosphate compounds (more exactly, the triesters of phosphoric or thiophosphoric acids) involve a group of highly toxic chemicals primarily used as pesticides (insecticides). Some extremely dangerous organophosphates are classified as nerve paralytic chemical warfare agents (sarin, soman, VX agent).^1^ The process of decontamination consists of the removal of hazardous materials from the contaminated surfaces by various physical, chemical or biological methods.^2^ For the decontamination of organophosphate compounds, chemical methods are still prevailing, although the alternative enzymatic routes have been extensively studied in recent times.^3^

Most of the conventional processes utilize rather aggressive chemicals that may damage the decontaminated surfaces and equipment. So-called “reactive sorbents” have been introduced as alternative decontamination agents capable of accumulating the toxic compounds on their surfaces and converting them into non-toxic products. The reactive sorbents consisting of nanocrystalline metal oxides or their composites were applied successfully to destroy the nerve agents, organophosphate pesticides^4^ and also some phosphorus-containing flame retardants.^5^ The metal oxide-based reactive sorbents are highly effective in non-polar and aprotic solvents, but (at least some of them) lose their efficiency in protic solvents and especially in aqueous media,^6^ which significantly limits their applications. Polymer-based materials, such as polyacrylonitrile or polyacrylamide particles with amidoxime functional groups, on the other hand, exhibited enhanced degradation efficiency towards CWAs in an aqueous dispersion.^7^ Zhang^8^ used magnetically separable magnetite nanoparticles covered with various copolymers and functionalized with amidoxime or hydroxamate functional groups for the destruction of some organophosphate pesticides (paraoxon) and other model compounds in aqueous solutions. The degradation efficiency of the amidoxime functionalized polyacrylonitrile fibres towards toxic organophosphates was strongly dependent on the presence of water.^9^ Recently, the oximated acrylate polymer was used successfully to destroy main nerve gases (sarin, soman and VX agent) as well as blistering agent sulfur mustard.^10^ In general, the oximate and hydroxamate groups known as strong nucleophilic agents^11^ are effective in the degradation of toxic organophosphates; their activity is influenced by the structure of the whole molecule^12^ and the matrix of the sorbent.^7^

Cellulose as a naturally occurring polymer, with its specific and versatile structure, offers some advantages (excellent hydrophilicity, biocompatibility) as a matrix for the reactive sorbents. As follows from the above-mentioned works, an introduction of the amidoxime functional group into the cellulosic matrix seems to be a reasonable strategy to prepare the reactive sorbent. It should be noted that similar kinds of the functionalized cellulose have been prepared by several authors, but they were used for the sorption of metal cations^13^ and its removal from electroplating wastewater,^14^ or for the extraction of uranium from seawater.^15,16^ Recently, Baek and Park^17^ developed a highly-porous amidoxime-functionalized cellulose-based sorbent with nearly uniform particles and tested it for the sorption of metal cations (Cu^2+^). However, to our knowledge, there is no application of the amidoxime-functionalized bead cellulose for the destruction of toxic organophosphates described in literature.

In this work, we used the commercially available regenerated cellulose for the preparation of the amidoxime-functionalized cellulose and demonstrated its ability to decompose the toxic organophosphates. The regenerated cellulose from the so-called TSGT (Thermal Sol–Gel Transition) process^18–21^ exhibits numerous beneficial properties useful for the advanced applications. In the TSGT process, the sol–gel transition is initiated by the temperature change under conditions resembling the homogeneous precipitation or block suspension polymerization, which results in the creation of uniform and porous spherical particles. The regenerated cellulose in the form of spherical particles is an attractive support for further derivatization and functionalization, which extends significantly its applications in separation sciences (chromatography), water treatment (metal-ion exchange), protein immobilization, solid-phase synthesis or targeted drug delivery.^22^

The AMD-functionalized bead cellulose was used successfully for the degradation of organophosphate pesticides (mainly parathion methyl) and toxic nerve gases (soman, VX agent) in aqueous media. Computational methods, quantum mechanics (QM) in the first place, were used to examine the mechanisms of the phosphoester cleavage.

## 2 Experimental

### 2.1 Preparation of the reactive sorbent

Regenerated cellulose supplied by Perloza Ltd. (Lovosice, Czech Republic) under the tradename Perloza ST was used for the preparation of the reactive sorbent. Acrylonitrile, hydroxylamine hydrochloride, ethanol (98%) and sodium hydroxide were purchased as reagent grade chemicals from Lach-Ner, Neratovice. The two-step synthetic route was used for the reactive sorbent preparation as follows: in the first step, the regenerated cellulose (450 g) was dispersed in ethanol and 136 g of acrylonitrile was added. The reaction mixture was agitated at 45 °C for 5 hours. The intermediate was separated by filtration and washed with ethanol and distilled water; it was stored in the form of an aqueous dispersion. Before its further use, the intermediate was homogenized, and the excessive water was removed by filtration. In the second step, the intermediate was re-dispersed (754 g) in the aqueous solution containing 208 g of hydroxylamine hydrochloride neutralized with 119.6 g NaOH. The reaction mixture was agitated at 65 °C for 8 hours. Finally, the functionalized cellulose was washed thoroughly with distilled water and stored in the form of an aqueous dispersion; it was further denoted as the AMD reactive sorbent.

### 2.2 Methods of characterization

The morphology of the samples was studied using an FEI Nova NanoSEM 450 scanning electron microscope with accelerating voltage 5–10  kV in a high-vacuum mode using circular backscatter (CBS) detector. The powder samples were placed onto carbon adhesive discs and sputter-coated by chromium thin layer (25 nm) before measurement. X-ray diffraction (XRD) analyses were performed with the aid of a PANalytical powder diffractometer X'pert PRO in symmetrical Bragg–Brentano configuration with CuKα radiation (<*λ*> = 1.5418 Å). X-ray photoelectron spectroscopy (XPS) measurements were performed using a SPECS PHIBOS 100 hemispherical analyser with a 5-channel detector and a SPECS XR50 X-ray source equipped with an Al and Mg dual anode. A Shirley background profile was used for data processing in CasaXPS software. Infrared spectra were recorded on Thermo Nicolet NEXUS 670 FTIR spectrometer in region 4000–500 cm^−1^ with the Praying Mantis (Harrick) diffuse reflection (DR) accessory.

### 2.3 Measurements of the degradation efficiency of the AMD reactive sorbent

Stock solutions of parathion methyl and paraoxon methyl with concentration of 40 mg L^−1^ were prepared by dissolving the respective analytical-grade chemicals in deionized water. TRIS buffers and acetate buffers with a concentration of 0.1 mol L^−1^ were prepared by dissolving Trizma base, Trizma hydrochloride solution, sodium acetate and hydrochloric acid in deionized water and mixing them at appropriate ratios. The chemicals were obtained from Sigma-Aldrich. Before the degradation experiment, the reactive sorbent was re-dispersed in deionized water, and an excess of water was removed by vacuum filtration. 1 g of the wet sorbent was placed into the Erlenmeyer flask and 40 mL of the solution containing 20 mg L^−1^ parathion methyl or paraoxon methyl and 0.05 mol L^−1^ of the buffer was added. The Erlenmeyer flask was agitated on the horizontal shaker with an intensity of agitation of 2 rpm in the air-conditioned room with the temperature of 22 ± 1 °C. In the predetermined time intervals, small portions of solution were taken by a pipette and analyzed by the liquid chromatographic system HPLC-DAD DIONEX UltiMate 3000 equipped with a diode array detector, 20 μL sampling loop, and a Poroshell 120-C18 2.7 μm column (Agilent, 50 mm × 3 mm). The methanol/water mixture was used as a mobile phase (both solvents contained 0.1% HCOOH) with a flow rate of 0.5 mL min^−1^, with a gradient starting from 40/60 to 90/10 (v/v).

Soman (*O*-pinacolyl methylphosphonofluoridate) and VX-agent (ethyl({2-[bis(propan-2-yl)amino]ethyl}sulfanyl)(methyl)phosphinate) were synthesized in the Military research institute. Their degradation was studied with the aid of the procedure used previously for the measurement of the degradation efficiency of the metal-oxide reactive sorbents:^23^ a portion of the wet reactive sorbent (typically 50 mg) was placed into a glass vial, and 1 mg of the toxic substrate was added on the layer of the homogenized sorbent. The vial was sealed with a cap and placed in a thermostat. All experiments were performed at 25 ± 1 °C. At the pre-determined time, the reaction was terminated by addition of *n*-hexane (1.85 mL), and the solid sorbent was separated from the suspension by centrifugation (14 500 rpm for 5 min). Several crystals of the water-free sodium sulphate were added to remove the residual water, and aliquots of the supernatant were analyzed by gas chromatography. The gas chromatograph was the Agilent 6890 system with an HP-5 column (5% phenyl methyl siloxane, 30 m × 0.32 mm ID × 0.25 μm film thickness) and a flame-ionization detector.

### 2.4 Safety precautions

Specifically trained personnel is required for manipulations with toxic organophosphates.

### 2.5 Computational methods

We have applied quantum chemistry (QM) methods together with some other computational methods to investigate the degradation mechanisms. Structure optimizations and transition state searches were performed using a minimal basis set method PBEh-3c,^34^ single-point calculations were performed using PBE0 hybrid DFT functional^35^ with dispersion corrections.^36,37^ Long-range solvent effects were included using the COSMO implicit solvation method.^38^ All calculations were performed using ORCA 4.2.1.^39,40^

## 3 Results and discussion

### 3.1 Characterization of the AMD reactive sorbent

**Fig. 1:**
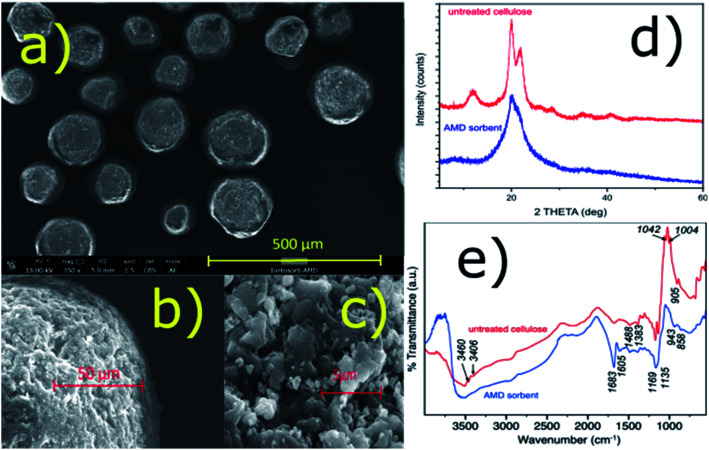
(a–c) SEM images of the particles of the AMD sorbent; (d) XRD patterns of the AMD sorbent and untreated bead cellulose; (e) FTIR spectra of the AMD sorbent and untreated bead cellulose.

It was found that the amidoxime-functionalized (AMD) sorbent retained most of the beneficial properties of the regenerated bead cellulose prepared by the TSGT process, such as morphology and enhanced mechanical resistance (low deformability) despite the fact that none (chemical) crosslinking was used.^18,24^ The sorbent consists of nearly uniform spherical particles with a complex inner structure – see the SEM images in Fig. 1a–c. The porosity of this kind of the bead cellulose typically ranges from 50–90%.^18^ For comparison, analogous SEM images of the underivatized bead cellulose are shown in ESI (Fig. S1†) demonstrating a great similarity in the morphology of the derivatized and underivatized bead cellulose. An aqueous dispersion of the AMD sorbent exhibits an excellent sedimentation behaviour and forms a compact bed with an exceptional permeability, when used in a column arrangement. The compact bed, however, can be easily re-dispersed in aqueous media, exhibiting advantageous rheological properties.

These unusual properties are results of the specific quaternary structure of the cellulosic polymer, in which the crystalline regions with a rigid structure stabilized by the hydrogen-bonding crosslinking are combined with amorphous regions,^18^ in accordance with the classical cellulose model.^25^ Using an X-ray diffraction, the presence of crystalline phase was confirmed together with a certain amount of an amorphous phase (Fig. 1c). A high degree of crystallinity of the bead cellulose (CI = 93%) was somewhat reduced after the derivatization (CI = 48% for the amidoxime functionalized cellulose). The crystallinity index (CI) was calculated by the peak-deconvolution method^26^ – for more details see ESI.† Certain decrease in crystallinity during the functionalization is related also to the reduction of the sizes of crystallites (from 6 to *ca.* 3 nm) in the functionalized cellulose.

IR spectra of untreated bead cellulose and AMD sorbent are presented in Fig. 1e. Some typical bands of cellulose structure were identified in the spectra of untreated sorbent. Namely, the broad peak centered at 3550 cm^−1^ with band at *ca.* 1670 cm^−1^ belong to OH stretching and bending of adsorbed water, while the small bands at 3460 and 3406 cm^−1^ were assigned to hydroxyl groups, respectively. The bands at 1488, 1383, together with several other small bands in the region 1383–1170 cm^−1^ are typical for various CH, CH_2_ and OH bending vibrations of crystalline cellulose. The intense sharp bands at 1169 and 1042 cm^−1^ belong to C–O–C, and C–O stretching, while the band at *ca.* 1139 cm^−1^ was assigned to ring antisymmetric stretching. The small but very typical band at around 905 cm^−1^ show existence of β-glycosidic linkage between glucose units in cellulose.^27–29^

Several significant changes were observed in the spectra of the AMD sorbent. The bands assigned to hydroxyl groups disappeared suggesting they are involved in formation of new functional groups upon modification. Furthermore, at the position of the water bending vibration (*ca.* 1670 cm^−1^), the sharp intense band and one smaller band were formed at 1683 and 1605 cm^−1^. The first was attributed to C=N stretching of amidoxime group^28,30^ while the latter remain unclear and was tentatively assigned to N–H band. The bands between 1488 and 1170 cm^−1^ practically remain undisturbed suggesting that some domains of the original crystal structure of the bead cellulose was maintained. On the other hand, the decrease of intensity of the bands at 1139, 1042 and 1004 cm^−1^ and the band at 905 cm^−1^ suggest some significant perturbance of the crystalline structure of the sorbent upon modification that is also consistent with XRD analysis. Finally, the band at 943 cm^−1^ can be assigned to N–O bond of the amidoxime group.^30^

**Fig. 2:**
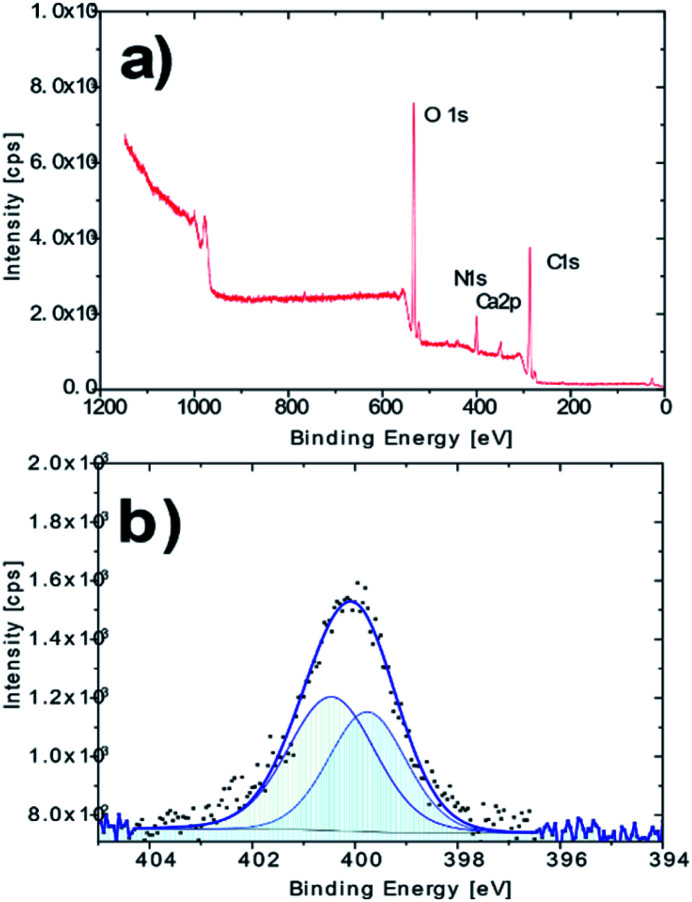
(a) XPS survey spectrum of the AMD sorbent; (b) high resolution N 1s spectrum of the AMD sorbent.

The XPS analysis confirmed the presence of nitrogen in the molecule of the derivatized cellulose in an amount of *ca.* 7.9%at (Fig. 2a). A more detailed XPS study showed characteristic changes in the high resolution O 1s and C 1s spectra, which may be associated with the newly created O–N and C–N bonds. The nitrogen N 1s high resolution spectrum on AMD cellulose shows a single peak at binding energy about 400.1 eV with extended FWHM about 2.2 eV, which indicates a possible presence of two components – N1 (=N–) at 399.8 eV and N2 (–NH_2_) at 400.5 eV, arising from the presence of the amidoxime group (Fig. 2b); the XPS spectra of the untreated cellulose together with some additional spectra of the AMD cellulose, the related discussion and literature references can be found in ESI.†

**Fig. 3:**
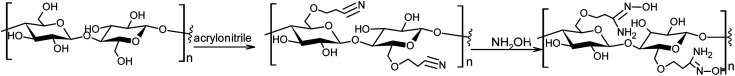
Scheme of the two-step preparation of the AMD sorbent.

An elemental analysis confirmed the presence of nitrogen in the functionalized sorbent. The contents of N, C and H were 9.37, 44.6 and 7.10%, respectively, which suggests that *ca.* 80% of the C-6 hydroxyl groups in the glucose units were converted to the amidoxime functional group according to the scheme shown in Fig. 3.

The presence of the nitrogen-containing functional group was confirmed also with the aid of thermogravimetric analysis – see ESI.† The content of the functional groups estimated from the metal-binding (Cu^2+^) capacity was 0.51 mmol g^−1^ (dry mass base), which agrees with the results of elemental analyses.

It was found that some key characteristics, such as morphology, crystallinity, elemental analysis and content of functional groups, as well as the degradation ability of the sorbent remained unchanged during the long-term (more than one year) storage in the form of the water dispersion.

### 3.2 Degradation of toxic organophosphates on the AMD reactive sorbent

**Fig. 4:**
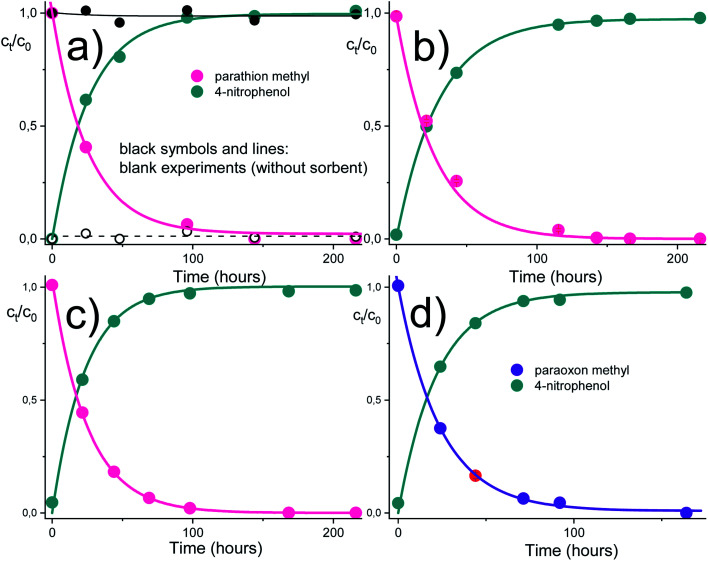
Time dependencies for the pesticide degradation in the presence of the AMD reactive sorbent. (a) parathion methyl, pH 5.02 (black symbols represent a blank experiment without sorbent, full circles – parathion methyl, open circles – 4-nitrophenol); (b) parathion methyl, pH 7.22; (c) parathion methyl, pH 9.00; (d) paraoxon methyl, pH 7.22. The initial pesticide concentration was 20 mg L^−1^, the buffer concentration was 0.05 mol L^−1^ and the concentration of sorbent was 12.5 g L^−1^.

The time dependencies for the pesticide degradation were measured as described in the experimental section. The HPLC method allowed to follow simultaneously both the pesticide removal as well as the creation of the degradation products. As can be seen from Fig. 4, both parathion methyl and paraoxon methyl were converted to 4-nitrophenol almost entirely in a time range of several hours. No other product or by-product (in addition to 4-nitrophenol) was observed during the degradation of parathion methyl and paraoxon methyl. It was also shown that almost identical degradation curves were obtained in acidic and basic solutions. More detailed examinations confirmed that the degradation of parathion methyl is only slightly affected by pH in a wide range up to *ca.* pH 10. This may be related to the acido-basic properties of the amidoxime functional group, which undergoes the protonation/dissociation equilibria (H_2_A^+^ ↔ HA ↔ A^−^), but the respective acidity constants differ markedly from each other (by several units on the log/pH scale).^31^ Therefore, only one form of the amidoxime group predominate over an almost whole range of experimental conditions (see ESI† for further considerations).

**Fig. 5:**
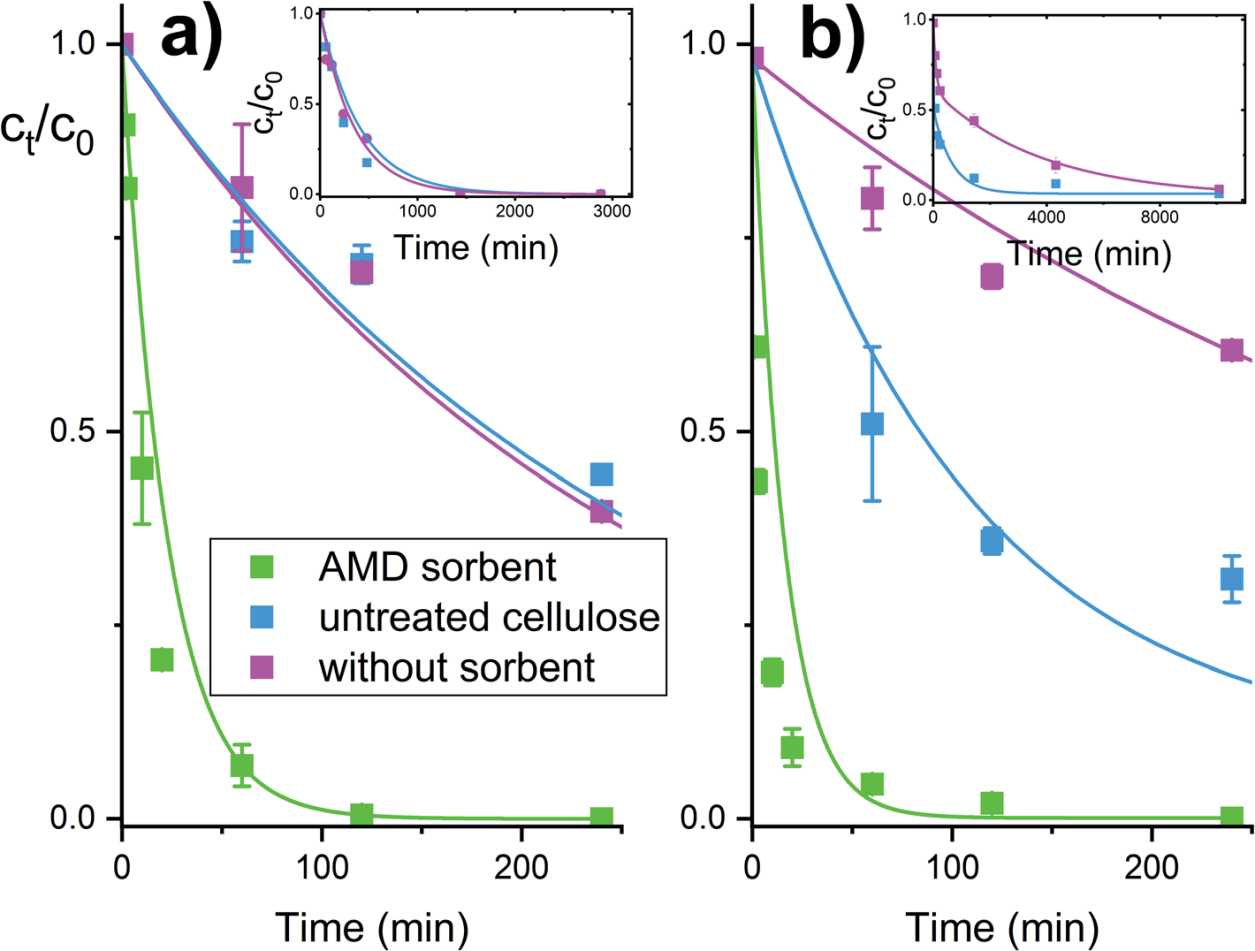
Degradation of nerve gases soman (a) and VX agent (b) in the presence of the AMD sorbent.

The reactive sorbent AMD also exhibited an ability to destroy some CWAs – soman and VX agent. These agents are unstable in aqueous media and are cleaved hydrolytically even in the absence of catalysts. In an aqueous dispersion of the AMD sorbent, then hydrolytic cleavage is combined with an accelerating effect of the amidoxime group, resulting in a very rapid degradation of these highly dangerous nerve gases (Fig. 5). Ethyl methylphosphonic acid (EMPA) and bis(S-2-diisopropyl amidoethane) (DES)_2_ are the product of the VX agent destruction on solid sorbents, whereas soman is cleaved to form methyl phosphonic acid, hydrogen fluoride and pinacolyl alcohol.^32,33^

The experimental dependencies in Fig. 4 and 5 were evaluated by the method of non-linear regression analysis with the aid of the pseudo-first order kinetic equations. Estimated model parameters are listed in Table 1.

**Table 1:** Kinetic parameters for the degradation of organophosphates (left part – disappearance of substrate, right part – creation of product). *q* is the organophosphate or 4-nitrophenol concentration in the time *t* divided by the initial concentration of organophosphate, *q*_∞_ is the residual fraction of organophosphate at the end of reaction, and *k*_obs_ is the overall (observed) rate constant

	*q* = e^−*k*_obs_*t*^ + *q*_∞_				*q* = 1 − e^−*k*_obs_*t*^		
	*k*_obs_ (h^−1^)	*t*_1/2_ (hours)	*q*_∞_	*R* ^2^	*k*_obs_ (h^−1^)	*t*_1/2_ (hours)	*R* ^2^
Parathion methyl, pH 5.02	0.038 (0.003)^a^	18.2	0.022 (0.011)	0.9960	0.035 (0.008)	18.3	0.9977
Parathion methyl, pH 7.22	0.025 (0.002)	27.7	0.002 (0.001)	0.9993	0.033 (0.004)	21.0	0.9960
Parathion methyl, pH 9.00	0.038 (0.003)	18.2	0.002 (0.001)	0.9999	0.043 (0.002)	16.0	0.9960
Paraoxon methyl, pH 7.22	0.041 (0.001)	16.9	0.011 (0.005)	0.9991	0.046 (0.003)	15.1	0.9959
Soman	N^b^	10^0^ to 10^1^ min	—	—	—	—	—
VX agent	N^b^	10^0^ to 10^1^ min	—	—	—	—	—

a Standard errors in parentheses.

b An exact estimation was not possible.

### 3.3 Mechanisms of the organophosphate degradation

Several mechanisms may be effective in the degradation of organophosphate compounds in the presence of the AMD reactive sorbent, including spontaneous hydrolytic cleavage of the phosphoester bond and nucleophilic substitution on the phosphorus atom.^8^ The computational methods provide a more in-depth insight into the mechanisms of the phosphoester cleavage. In ESI,† a more detailed study is given on the hydrolysis of several pesticides and chemical warfare agents.

It was assumed that the hydrolysis of organophosphate pesticides follows a two-step mechanism, similar to that previously reported for paraoxon methyl.^41^ The first step of the reaction, associated with the transition state TS1, is an attack of the water molecule opposite of the phosphoester bond with a simultaneous proton transfer from the attacking water onto the axial sulphur or oxygen atom, which results in a pentacoordinate phosphorane intermediate state INT. The second step of the reaction is the breakage of the phosphoester bond and a proton transfer from the axial sulphur or oxygen to the leaving oxygen atom of the original phosphoester bond.

The methodology was first applied to study of the hydrolysis of several organophosphate pesticides in water. The calculated values of the reaction energetic barriers were successfully correlated with some previously published experimental data – namely with the rate constants obtained by Kamiya *et al.*^42^ for the hydrolytic cleavage of paraoxons and parathions, and with the activation energies of the pesticide degradation in the methanol–water mixture;^43^ see ESI for more details.† Excellent correlations between the calculated values and experimental data prove that the computational methods used are applicable to study the organophosphate degradation in real systems.

**Fig. 6:**
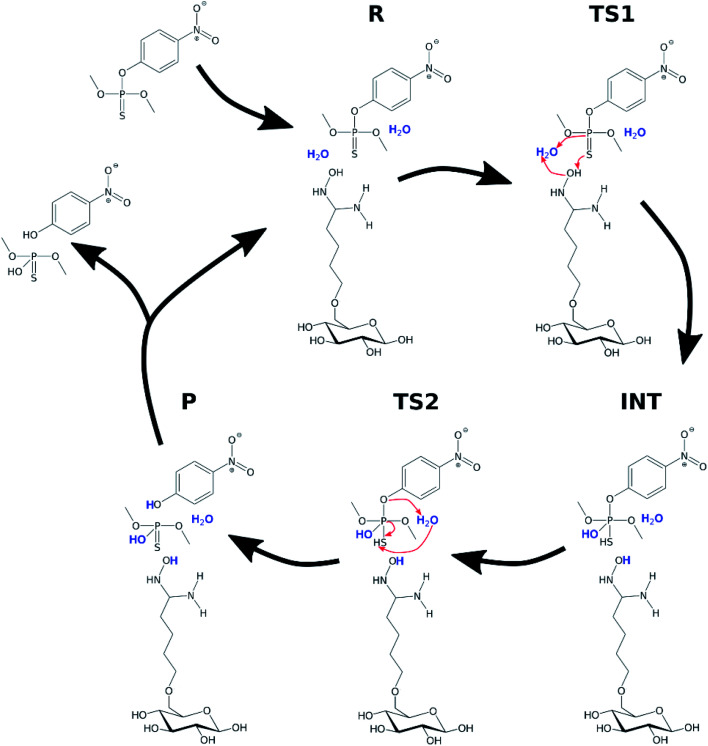
Degradation of parathion methyl in the presence of the AMD sorbent. Water molecules participating in the reaction are highlighted.

In order to investigate the organophosphate degradation catalyzed by amidoxime-functionalized cellulose, a simplified model was considered. The model consisted of a single glucose molecule functionalized with amidoxime at the C6 position. In this part of the study, parathion methyl and VX agent were selected as representatives of organophosphate pesticides and CWAs, respectively. A conventional molecular dynamics simulation of the functionalized glucose with a single parathion methyl molecule was performed to obtain a mutual orientation of these molecules. Simulation details are provided in ESI.† Obtained structure was used for the subsequent QM calculations. The reaction mechanism was assumed to be similar to the hydrolysis in water with the oxime group activating an attacking water molecule *via* hydrogen withdrawal. The scheme of the reaction is shown in Fig. 6.

Two explicit water molecules were included with the modified glucose and parathion methyl. Long-range solvent effects were included using the COSMO implicit solvation method.^38^ Initial structures (R, TS1, INT, TS2, P) were constructed based on the corresponding structures from the hydrolysis in water; they were then optimized using PBEh-3c and single-point energies calculated using PBE0, same as in previous calculations.

The relative potential reaction energy, intermediate state energy and energy barriers associated with the two transition states are shown in Table 2. As can be seen, the barrier of the first and rate-limiting step (TS1) was reduced by 20 kcal mol^−1^ to 11.37 kcal mol^−1^ (36% of the original barrier). If we consider the energy of the reaction barrier to be equal to the activation energy, then a reduction by 20 kcal mol^−1^ would mean a multiplication of the rate constant by a coefficient of about 10^14^. Although this may be considered not entirely correct interpretation, there are no doubts that the reduced reaction barrier leads to a significant enhancement of reactivity of parathion methyl in the presence of the amidoxime functional group. The energy values of the intermediate (INT) state and the second reaction barrier are also reduced compared to the water hydrolysis. The difference in the energy between INT state and the second barrier is smaller than the uncertainty of the computational method, which indicates that the intermediate state may no longer be relevant in the oxime-catalyzed hydrolysis.

**Table 2:** Potential energies relative to the reactant state (*R*) in kcal mol^−1^ calculated at the PBE0 level

	Parathion methyl	Parathion methyl + AMD	VX	VX + AMD
**TS1**	**31.40**	**11.37**	**17.79**	**12.88**
INT	20.26	8.90	—	—
TS2	26.57	8.93	—	—
P	−16.79	−5.02	−17.80	−11.19

**Fig. 7:**
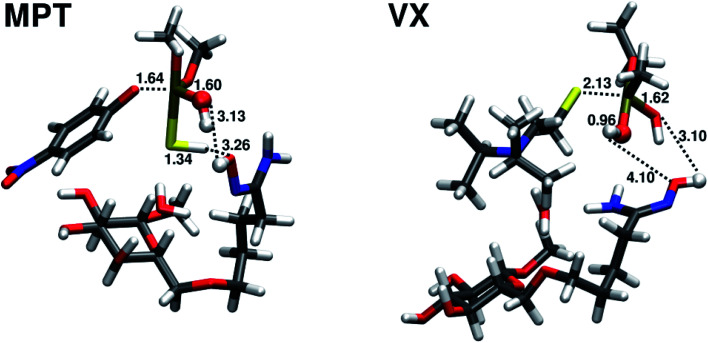
Transition states TS1 for the amidoxime-catalysed hydrolysis of parathion methyl (MPT) and VX. Distances (in Å) of atoms involved in the attacking, leaving bonds and the proton transfer are shown. Atoms of the attacking water molecule are shown as balls.

The transition state TS1 of the amidoxime-catalyzed hydrolysis of parathion methyl is shown in Fig. 7. The transition state is similar to the transition state in the water hydrolysis, but with shorter distances for both the leaving oxygen and the attacking oxygen.

The orientation of parathion methyl molecule and modified glucose was used to construct the complex of VX and the modified glucose. Two explicit water molecules were included with the modified glucose and VX. Initial structures (R, TS, P) were constructed based on the corresponding structures from the hydrolysis in water and same computational methodology was used for their optimization.

The hydrolysis of VX does not follow the two-step mechanism of the other organophosphates (see ESI†). Same was assumed for the amidoxime-catalyzed hydrolysis of VX. The relative reaction energy and reaction barrier are given in Table 2. The reaction barrier of the amidoxime-catalyzed hydrolysis compared to water hydrolysis is reduced by almost 5 kcal mol^−1^ to 12.88 kcal mol^−1^ (72% of the original barrier). This reduction in the barrier height is much less pronounced than in the case of parathion methyl. The transition state of the amidoxime-catalyzed hydrolysis of VX is shown in Fig. 7.

## 4 Conclusions

A simple two-step synthesis was used to introduce the amidoxime functional group into the cellulosic matrix. The functionalized cellulose retained beneficial properties of the pristine bead cellulose, such a highly hydrophilic nature, mechanical resistance, good dispersibility, permeability and beneficial rheological properties favouring its use in column arrangements. It was shown that the functionalized cellulose significantly accelerates the destruction of highly toxic organophosphate compounds, including organophosphate pesticides and nerve gases (soman, VX agent). Computer simulations, including QM calculations were used to study the degradation mechanisms at the molecular level.

In the case of the amidoxime-stimulated destruction of parathion methyl, the two-step mechanism is effective, consisting of a nucleophilic attack to the phosphorus atom by the water molecule activated by the amidoxime group *via* hydrogen withdrawal. The reaction goes through two transition states and a pentacoordinate phosphorane intermediate. In the second step, the phosphoester bond is broken, followed by the proton transfer from the sulphur atom to the oxygen atom in the leaving group. It follows from the QM calculations that the reaction energy barrier in the first (rate-limiting) step is reduced significantly in the presence of the AMD reactive sorbent. In accordance with this prediction, the respective rate constant increased markedly resulting in a quick destruction of this pesticide.

The amidoxime-catalyzed destruction of the VX agent proceeds *via* somewhat different (one-step) reaction scheme, and the reduction in the reaction energy barrier is less pronounced than in the case of parathion methyl. Apparently (relatively), the catalytic effect of the amidoxime group is less significant, as other mechanisms (hydrolytic cleavage) are effective simultaneously. It is essential from the practical point of view that the AMD reactive sorbent is capable of destroying hazardous nerve agents within several minutes, and thus belongs to the most effective decontamination agents available for those purposes. Bead cellulose as a readily available and environmentally compatible material with well-defined mechanical properties and good chemical reactivity is a promising platform for a new generation of the cellulose-based sorbents.

## Author contributions

Pavel Janoš: conceptualization, supervision, methodology, investigation, writing – original draft, supervision, project administration, funding acquisition. Oldřich Tokar: methodology, validation, investigation, resources, data curation. Marek Došek: investigation, data curation, visualization. Karel Mazanec: methodology, validation, investigation, resources, funding acquisition. Petr Ryšánek: investigation, methodology, data curation. Martin Kormunda: investigation, methodology, data curation. Jiří Henych: methodology, validation, investigation, writing-reviewing and editing. Pavel Janoš, Jr.: conceptualization, methodology, validation, investigation, writing – original draft, writing – reviewing and editing.

## Conflicts of interest

There are no conflicts to declare.

## Supplementary Material

RA-011-D1RA01125A-s001
