# Supplementary material for: Amidoxime-functionalized bead cellulose for the decomposition of highly toxic organophosphates
Source: RSC Adv. 2021 May 18;11(29):17976–84. doi: 10.1039/d1ra01125a (PMC9033230; doi:10.1039/d1ra01125a)
Supplement: RA-011-D1RA01125A-s001 [file RA-011-D1RA01125A-s001.pdf]

Electronic Supplementary Material (ESI) for RSC Advances.
This journal is © The Royal Society of Chemistry 2021

Amidoxime-functionalized bead cellulose for the decomposition of highly toxic organophosphates

Pavel Janoš*, Oldřich Tokar, Marek Došek, Karel Mazanec, Petr Ryšánek, Martin Kormunda, Jiří Henych, Pavel Janoš, Jr.

Electronic Supplementary Information

Content

S.1. SEM images of the bead cellulose 1
S2 X-ray diffraction and XPS analysis 2
S.3. Thermogravimetric analysis 6
S.4. Reaction kinetics 6
S.5. Degradation of toxic organophosphates – a computational approach 8

S.1. SEM images of the bead cellulose

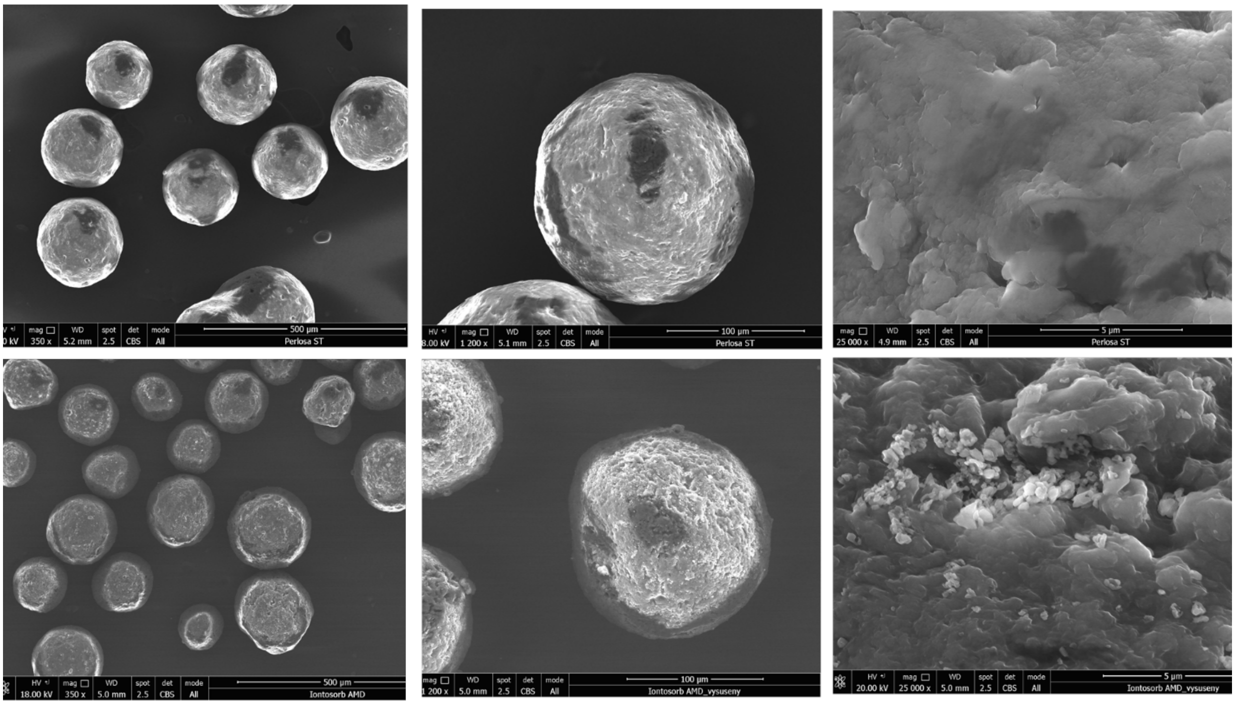

Fig. S1. SEM images of unfunctionalized bead cellulose (top row) and amidoxime-functionalized bead cellulose (bottom row).

S2 X-ray diffraction and XPS analysis

The crystallinity of the cellulosic samples was estimated by the peak-deconvolution method under an assumption that the amorphous cellulose is the main contributor to the peak broadening [1]. Then the index of crystallinity may be calculated from areas of the crystalline peaks (blue, grey, and brown line in the following figure) divided by the total peak area.

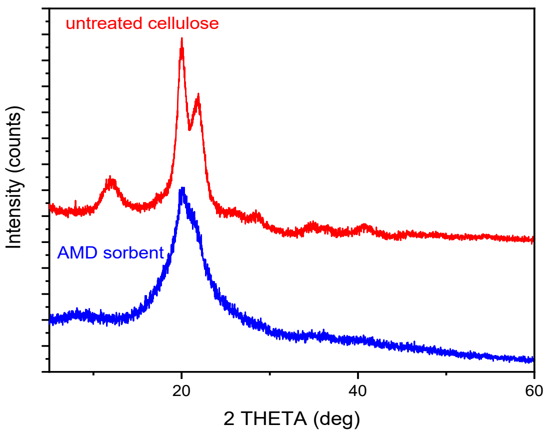

Fig. S2. XRD diffraction patterns of the untreated cellulose and AMD sorbent

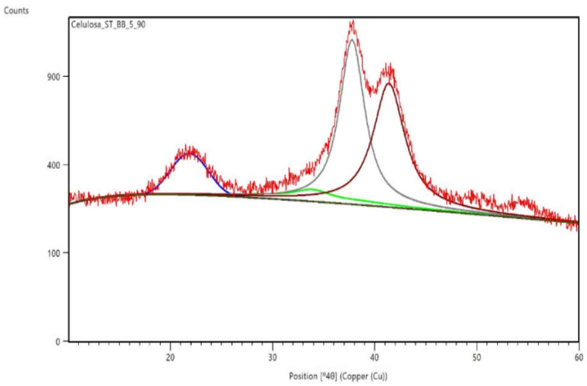

Fig. S3. Determination of the cellulose crystallinity from the XRD patterns using the peak deconvolution method. The crystallinity was calculated from the sum of areas of the peaks corresponding to the crystalline phases divided by the total peak area after the background subtraction.

The XPS measurements confirmed the presence of carbon and oxygen in the ratio of 64/36 in the untreated cellulose (Fig S4a); the high resolution spectrum was identical to that published in literature [2]. Pure cellulose exhibits two peaks in carbon C1s XPS spectra attributed to C-O (alcohols and ethers) and O-C-O (acetal) moieties. In practice, the XPS analysis of cellulose reveals three C1s peaks at 285.0, 286.8 and 288.4 eV, arising from components C1 (C-C/C-H), C2 (C-O) and C3 (O-C-O and/or C=O), respectively [2,3], see Fig. S5a. The corresponding oxygen O1s XPS spectra are rarely presented in literature - they reveal two components O1s the main 533.3 eV and minor 351.1 eV, arising from components O1 (O-C) and O2 (O=C), see in Fig. S6a.

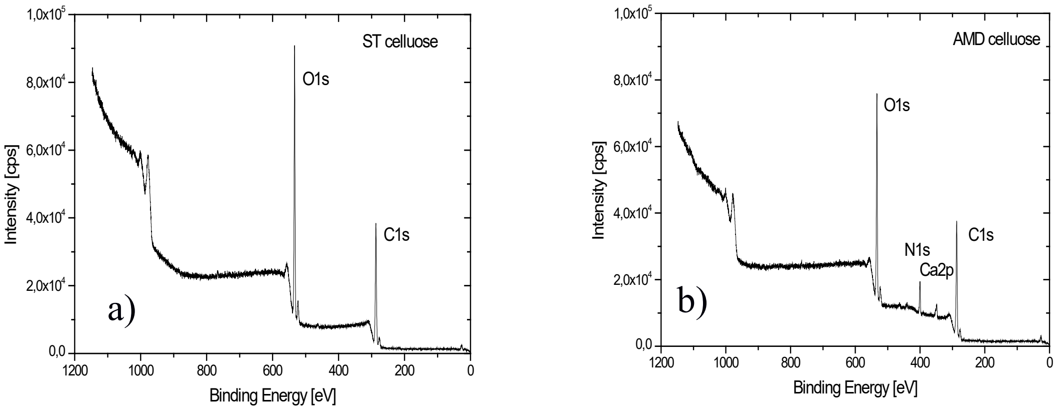

Fig. S4. XPS survey spectra of untreated cellulose (a) and AMD sorbent (b)

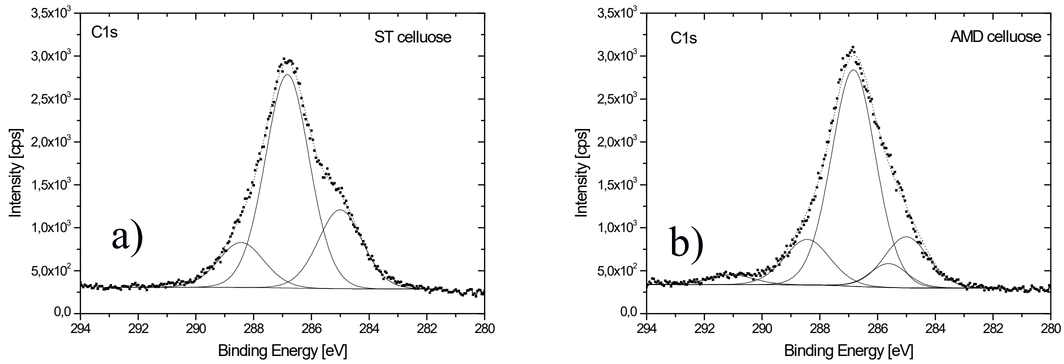

Fig. S5. High resolution XPS spectra of carbon C1s of untreated cellulose (a) and AMD sorbent (b)

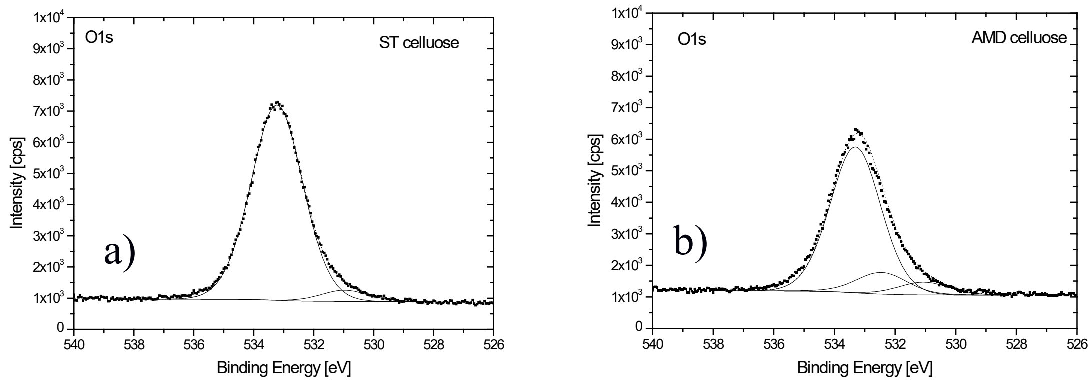

Fig. S6. High resolution XPS spectra of carbon O1s of untreated cellulose (a) and AMD sorbent (b)

The modified AMD cellulose shows additional nitrogen content about 7.9 at.% and small calcium content about 1.2 at.%. (Fig. S4b). The high resolution XPS spectra of carbon C1s have similar shapes to spectra observed on the unmodified cellulose (Fig. S5) with the different shape at the low energy side where a number of C-C bonds is reduced, and an additional small component C4 at 291.1 eV has to be introduced. Therefore the C4 component should be associated to some of C to oxygen bonds, for example C(=O)O [3]. The C-N bonds mostly overlaps C-O bonds and identification of individual bonds is unsure but an additional component at 285.6 eV was be introduced to fit the spectrum of AMD cellulose; the C5 component is associated to some of C to N bonds - for example C–NH2, the part of the amidoxime group.

The oxygen O1s peak in AMD cellulose exhibits some changes; the component O2 related to O=C bonds at 351 eV is increased and new component O3 related to 2NH-C=N-OH structure at 352.45 eV [3] appeared (Fig. S6).

The nitrogen N1s high resolution spectrum on AMD cellulose shows a single peak at binding energy about 400.1 eV with higher FWHM about 2.2eV, which indicates a possible presence of two components. The XPS studies of amidoxime related materials are available but the components of the N1s peak are reported controversially, as summarized in Table S1.

We have observed in AMD cellulose the N1s spectrum two components; first N1 at 399.8 eV and second N2 at 400.5 eV, arising from the presence of the amidoxime. The amidoxime related components are in the ratio N1/N2 = 44/56. Moreover, we also identified additional amidoxime related components in C1s and O1s spectra.

Table S1: Literature survey study: XPS N1s spectra of the amidoxime group

a-N1s (=N-)	b-N1s (-NH2)	(a-b)	C1s (C-C/C-H)	Cit.
398.37 eV	398.82 eV	-0.45 eV	285.36 eV	[4]
400.0 eV	399.6 eV	0.4 eV	284.6 eV	[5]
399.0 eV	399.6 eV	-0.6 eV	unknown	[6]
398.5 eV	399.4 eV	-0.9 eV	284.2 eV	[7]
400.4 eV	399.4 eV	1 eV	284.8 eV	[8]
400.2 eV	399.5 eV	0.7 eV	285.0 eV	[9]
400.46 eV	399.52 eV	0.94 eV	284.64 eV	[10]

References

[1] S. Park, J.O. Baker, M.E. Himmel, P.A. Parilla, D.K. Johnson, Cellulose crystallinity index: Measurement techniques and their impact on interpreting cellulase performance, Biotechnol. Biofuels. 3 (2010) 10. https://doi.org/10.1186/1754-6834-3-10.

[2] M.N. Belgacem, G. Czeremuszkin, S. Sapieha, A. Gandini, Surface by XPS characterization and inverse gas of cellulose fibres chromatography, 1995.

[3] J. Ederer, P. Janoš, P. Ecorchard, J. Tolasz, V. Štengl, H. Beneš, M. Perchacz, O. Pop-Georgievski, Determination of amino groups on functionalized graphene oxide for polyurethane nanomaterials: XPS quantitation vs. functional speciation, RSC Adv. 7 (2017) 12464–12473. https://doi.org/10.1039/C6RA28745J.

[4] F. Ma, B. Dong, Y. Gui, M. Cao, L. Han, C. Jiao, H. Lv, J. Hou, Y. Xue, Adsorption of Low-Concentration Uranyl Ion by Amidoxime Polyacrylonitrile Fibers, Ind. & Eng. Chem. Res. 57 (2018) 17384–17393. https://doi.org/10.1021/acs.iecr.8b03509.

[5] B. Yu, L. Zhang, G. Ye, Q. Liu, J. Li, X. Wang, J. Chen, S. Xu, S. Ma, De novo synthesis of bifunctional conjugated microporous polymers for synergistic coordination mediated uranium entrapment, Nano Res. 14 (2021) 788–796. https://doi.org/10.1007/s12274-020-3217-7.

[6] S. Zhuang, J. Wang, Poly amidoxime functionalized carbon nanotube as an efficient adsorbent for removal of uranium from aqueous solution, J. Mol. Liq. 319 (2020) 114288. https://doi.org/10.1016/j.molliq.2020.114288.

[7] J. Xiao, Y. Jing, X. Wang, Y. Yao, Y. Jia, Preconcentration of Uranium(VI) from Aqueous Solution by Amidoxime-Functionalized Microspheres Silica Material: Kinetics, Isotherm and Mechanism Study, ChemistrySelect. 3 (2018) 12346–12356. https://doi.org/10.1002/slct.201802472.

[8] X. Yin, J. Bai, W. Tian, S. Li, J. Wang, X. Wu, Y. Wang, F. Fan, Q. Huang, Z. Qin, Uranium sorption from saline lake brine by amidoximated silica, J. Radioanal. Nucl. Chem. 313 (2017) 113–121. https://doi.org/10.1007/s10967-017-5283-1.

[9] J. Shen, W. Huang, L. Wu, Y. Hu, M. Ye, Study on amino-functionalized multiwalled carbon nanotubes, Mater. Sci. Eng. A. 464 (2007) 151–156. https://doi.org/10.1016/j.msea.2007.02.091.

[10] J. Xiao, Y. Jing, Y. Yao, X. Wang, Y. Jia, Synthesis of amidoxime-decorated 3D cubic mesoporous silica via self-assembly co-condensation as a superior uranium(VI) adsorbent, J. Mol. Liq. 277 (2019) 843–855. https://doi.org/10.1016/j.molliq.2019.01.009.

S.3. Thermogravimetric analysis

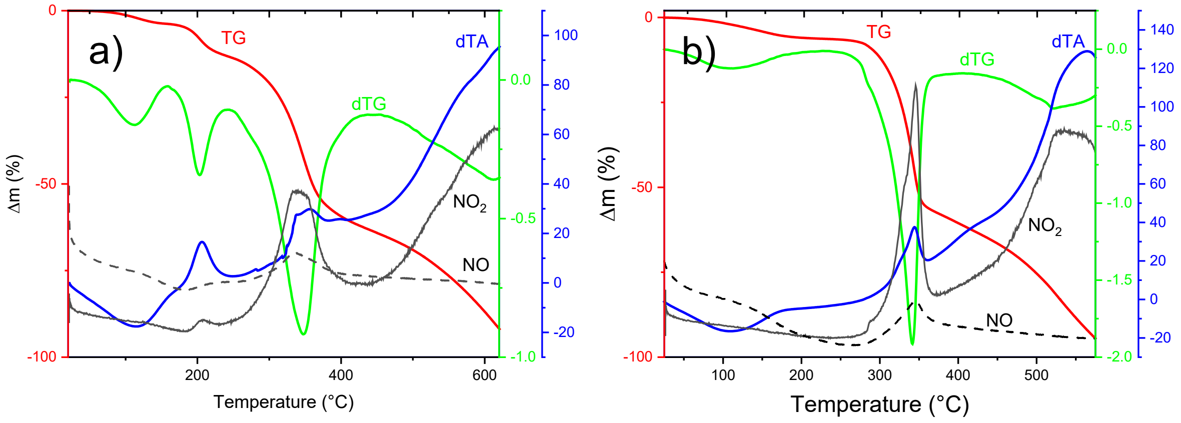

Fig. S7. Thermogravimetric analyses of the amidoximated cellulose (a) and the untreated bead cellulose (b). Apparatus: SETARAM Instr., crucible Al_2O_3, carrier gas: air. Black lines - MS determination of NO_2 (full line) and NO (dashed line).

S.4. Reaction kinetics

It is assumed that the degradation of organophosphates may be described by a simple (overall) equation: A →B. For example, parathion methyl is converted to 4-nitrophenol. However, even in this simplified case, several mechanisms may be effective:

a) Spontaneous (autocatalytic) decomposition, which can be described by an equation:

$$-\frac{d[A]}{dt} = k_S[A] \quad (1)$$

b) Catalytic activation with amidoxime group. Here, we assume that this group undergoes dissociation/protonation in dependence on the pH value; in slightly acidic to basic media, the dissociation is assumed:

$$[HCat] \Leftrightarrow [H^+] + [Cat^-]; \qquad K_a = \frac{[H^+][Cat^-]}{[HCat]} \quad (2)$$

Both the dissociated and undissociated form may exhibit the catalytic activity:

$$-\frac{d[A]}{dt} = k_{C1}[A][HCat] \quad (3)$$

$$-\frac{d[A]}{dt} = k_{C2}[A][Cat^-] \quad (4)$$

c) Hydrolytic cleavage by (presumably) the OH^- groups:

$$-\frac{d[A]}{dt} = k_{OH}[A][OH^-] \qquad (5)$$

The overall reaction rate can be written as

$$-\frac{d[A]}{dt} = k_{obs}[A] \qquad (6)$$

which gives

$$\frac{[A]}{[A_0]} = e^{-k_{obs}t} \qquad (7)$$

where

$$k_{obs} = k_S + \frac{k_{C1}[H^+]c_{CAT}}{[H^+]+K_a} + \frac{k_{C2}K_a c_{CAT}}{[H^+]+K_a} + \frac{k_{OH}K_w}{[H^+]} \qquad (8)$$

K_w is ionic product of water and c_{CAT} is the concentration of catalyst. A similar set of equations can be written for the product creation.

Eq. (8) presents the pH dependence of the overall (observed) rate constant. Unfortunately, most of the model parameters, such as values of individual rate constants, are not known. Effects of some parameters on the pH dependence of the rate constant were modelled using Eq. (8), and are visualized in the following figures:

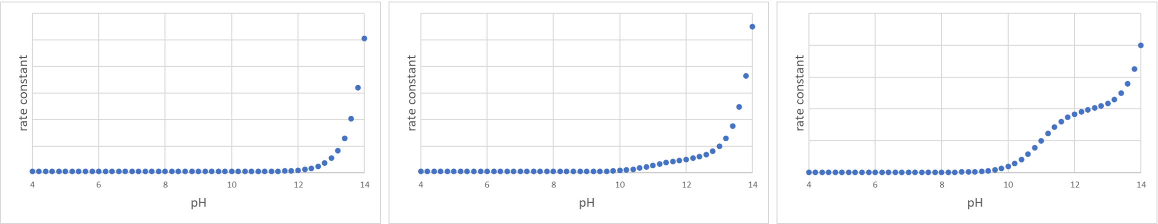

Fig. S8. Effect of the activity of catalyst on the overall rate constant. The catalytic activity increases in the ratio 1 : 10 : 100 from the left to right.

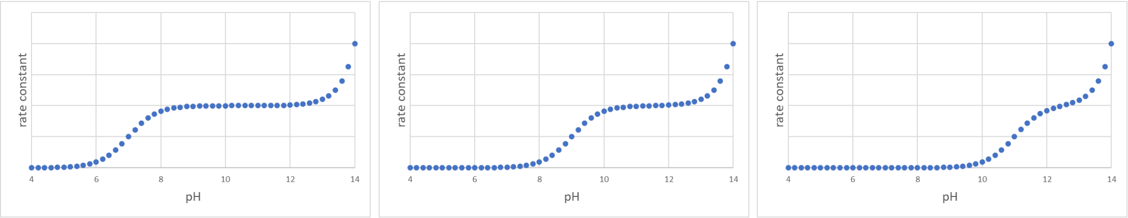

Fig. S9. Effect of the pK_a value of the amidoxime functional group on the overall rate constant. The pKa values are 7; 9, and 11, respectively, from the left to right.

In fact, the amidoxime functional group can be considered dibasic with pK_a values quite distant from each other, and with the second pK_a value laying in alkaline area. For example, for acetamidoxime, the respective pK_a values are 5.78 and 13.21, and for benzamidoxime 4.85 and

12.36, respectively [1]. It can be assumed that the activity of the amidoxime functional group is not affected by the pH value in a broad range.

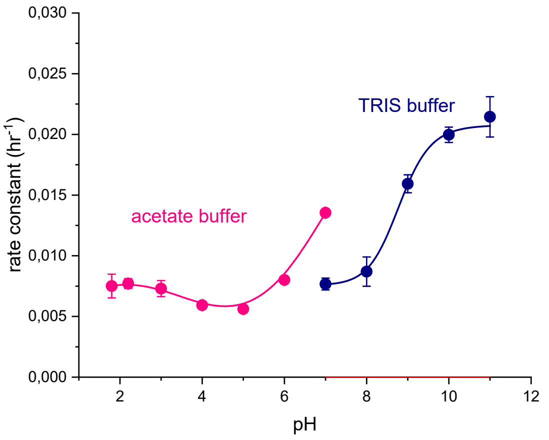

Fig. S10. Experimental dependence of the degradation efficiency on pH

Reference

[1] N. Mehio, M. A. Lashely, J. W. Nugent, L. Tucker, B. Correira, C.-L. Do-Thanh, R. D Hanckock, V. S. Bryantsev. Acidity of the amidoxime functional group in aqueous solution: A combined experimental and computational study. J. Phys. Chem. B, 119 (8), 3567-3576 (2015).

S.5. Degradation of toxic organophosphates – a computational approach

In the first part of the study, we applied quantum mechanics (QM) calculation methods to investigate the hydrolytic cleavage of several toxic organophosphates in pure water. The reaction path of the hydrolysis was taken from the theoretical investigations of Chagas et al. [1] on the hydrolysis of paraoxon ethyl. We applied this approach to several organophosphate pesticides – namely to parathion methyl, parathion ethyl, paraoxon methyl, paraoxon ethyl, and EPN (O-ethyl O-(4-nitrophenyl) phenylphosphonothioate – and to VX agent as a representative of nerve paralytic chemical warfare agents (CWAs). Structure optimizations and transition state searches were performed using minimal basis set method PBEh-3c, which provides high quality structures within a reasonable computational time [2]. Single point calculations were performed using PBE0 hybrid DFT functional [3] with D-3 dispersion corrections [4,5]. Three water molecules, that participate in the hydrolysis, were explicitly included with each organophosphate. Long range solvent effects were included using the COSMO implicit solvation method [6]. All calculations were performed using ORCA 4.2.1 [7,8].

Hydrolysis of organophosphate pesticides

The hydrolysis of organophosphate pesticides was assumed to follow the two-step mechanism reported in [1] for paraoxon ethyl. The reaction is schematically shown in Fig. S11.

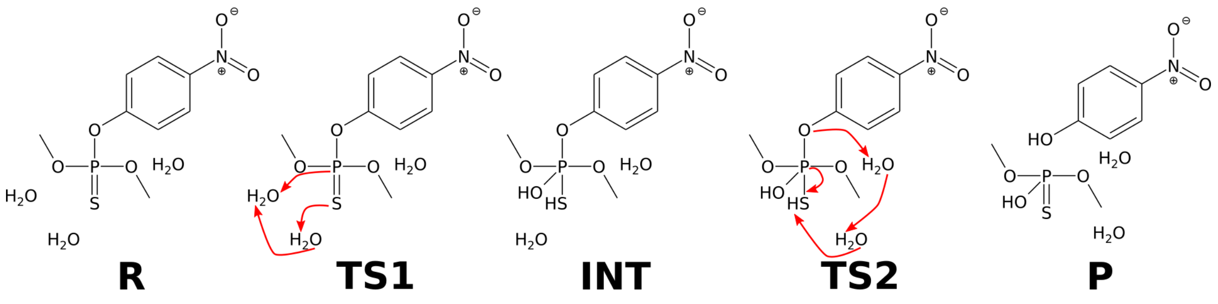

Fig. S11. Scheme of the neutral water hydrolysis of parathion methyl.

The hydrolytic reaction is initiated by an attack of the water molecule opposite to the phosphoester bond accompanied by a proton transfer from the attacking water molecule onto the axial sulphur or oxygen atom; the first step of the reaction is associated with the transition state TS1. This results in a pentacoordinate phosphorane intermediate state INT. The second step of the reaction is the breakage of the phosphoester bond and a proton transfer from the axial sulphur or oxygen to the leaving oxygen atom of the original phosphoester bond. The structures of parathion methyl associated with all the stages of the reaction were taken from [1] and used as a basis for the hydrolysis simulations of other organophosphate pesticides. In all cases three explicit water molecules were included with the molecule of the organophosphate. Geometry optimizations, and transition state searches, were performed using the methodology described above.

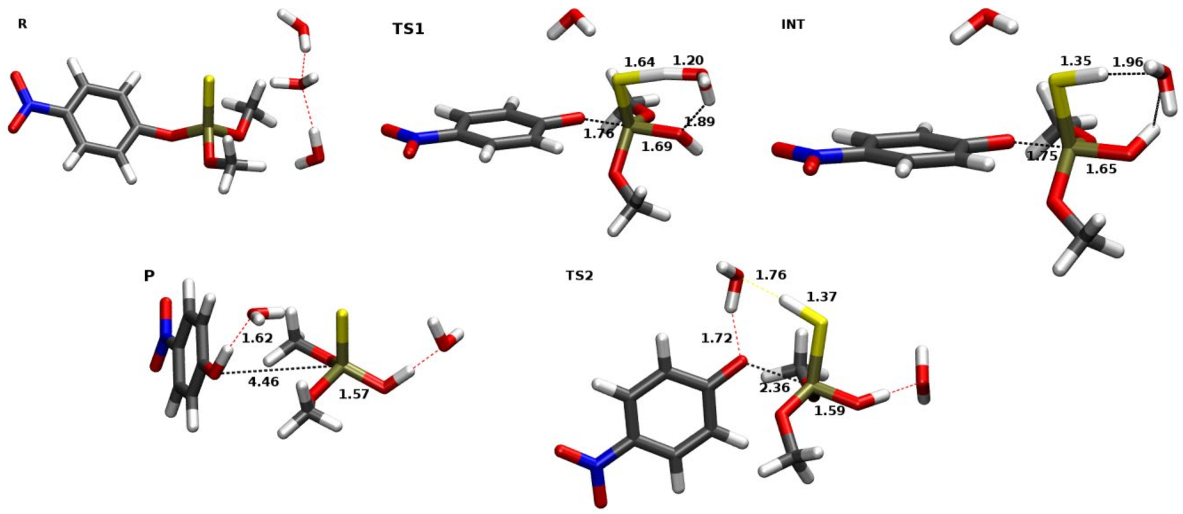

Fig. S12. Hydrolysis of parathion methyl – structures of reactant (R), transition states (TS1, TS2), intermediate (INT) and product (P).

Fig. S12 shows individual structures occurring during the parathion methyl hydrolysis. Table S2 and Fig. S13 summarize the results for all the studied compounds and show the relative potential reaction energies, intermediate energies and energy barriers associated with the two transition states. The results (the energy barriers and reaction energy) obtained for paraoxon ethyl are consistent with the published values [1].

It follows from the simulations that the assumed two-step reaction mechanism is valid for all pesticides with an exception of paraoxon methyl and EPN. In the case of EPN the second transition state has the same energy as the intermediate state, while in the case of paraoxon methyl the second transition state is even lower in energy than the intermediate. However, the magnitude of the difference is comparable with an uncertainty of the used method. In the other cases the reaction barrier of the second step is lower that the barrier of the first step. Thus, the reaction barrier TS1 represents the main reaction barrier and the first step is the rate-limiting step of the hydrolysis reaction. Among the examined pesticides, the parathions have larger energy barriers than paraoxons, and EPN has the lowest energy barrier of all the studied organophosphate pesticides.

Table S2. *Potential energies relative to the reactant state (R) in kcal/mol calculated at the PBE0 level*

	paraoxon methyl	paraoxon ethyl	parathion methyl	parathion ethyl	EPN
**TS1**	**27.15**	**27.95**	**31.40**	**32.89**	**21.04**
INT	13.95	15.36	20.26	21.97	19.64
TS2	13.47	17.67	26.57	26.97	19.64
P	-15.33	-15.52	-16.79	-16.58	-11.60

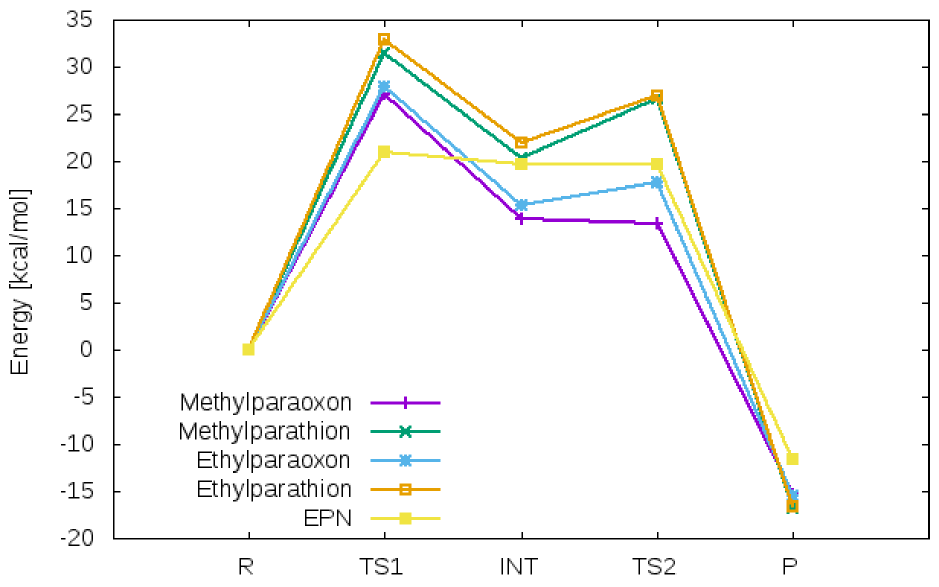

Fig. S13. Potential energies during the pesticide hydrolysis relative to the respective reactant state (R) in kcal/mol calculated at the PBE0 level.

Table S3. *Partial atomic charge on the phosphorus atom for studied organophosphate pesticides at individual stages of the hydrolysis reaction*

	paraoxon methyl	paraoxon ethyl	parathion methyl	parathion ethyl	EPN
R	0.8525	0.8280	0.6888	0.6873	0.5942
TS1	0.9477	0.9310	0.8111	0.7966	0.6877
INT	0.8976	0.8870	0.7829	0.7701	0.6770
TS2	0.9122	0.9333	0.8178	0.8092	0.6770
P	0.8563	0.8497	0.7160	0.7014	0.5866

The energy barriers listed in Table S2 can be used to predict the kinetics of the pesticide hydrolytic cleavage.

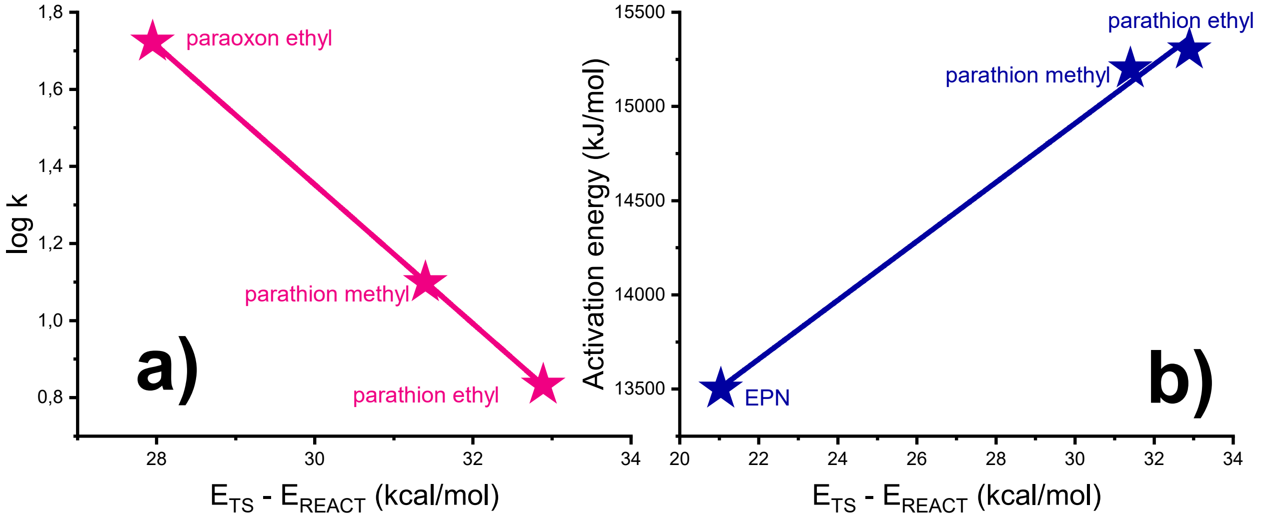

Fig. S14. a) Correlation between the calculated energy barrier and the pseudo-first order rate constants of the hydrolysis of some pesticides in phosphate buffer, pH 8.5 (9); b) correlation between the calculated energy barrier and the activation energy determined from experimental data for the hydrolysis of pesticides in the methanol-water mixed solvent [10].

We attempted to correlate the computed results with some experimental data from literature. However, although the pesticide hydrolysis has been frequently studied in various systems and arrangements, it is difficult to find a consistent set of quantitative data on the reaction kinetics. Data for the hydrolytic cleavage of paraoxons and parathions were found in the study of Kamiya et al. [9]. As can be seen from Fig. S14a), the values of the rate constants may be correlated with the calculated values of the energy barriers using the Arrhenius equation. Sato and Kubo [10] determined experimentally the activation energies for the pesticide degradation in the methanol-water mixture. As can be seen, these values also correlate well with the reaction

barriers calculated in this work. Excellent correlations between the calculated values and experimental data prove that the computational methods used are applicable to study the organophosphate degradation in real systems.

Fig. S15 shows the transitions states TS1 associated with the main reaction barrier for all pesticide substrates. The distance between the leaving oxygen and the phosphor atom is consistently 1.75 Å – 1.77 Å across all pesticides. However, the distance between the attacking oxygen atom and the phosphor atom differs between the paraoxons and parathions - the distance is shorter in parathions (1.69 Å) then in the paraoxons (1.80 Å). The proton transfer via a water molecule is almost already completed in the case of parathions, while in paraoxons the proton from attacking water is not yet fully transferred to the secondary/auxiliary water molecule. The EPT, which shares the sulphur atom in axial position with parathions, has transition state similar to the parathions with proton transfer already completed. Indicating that the TS1 in sulphur-containing OPs is perhaps a late transition state.

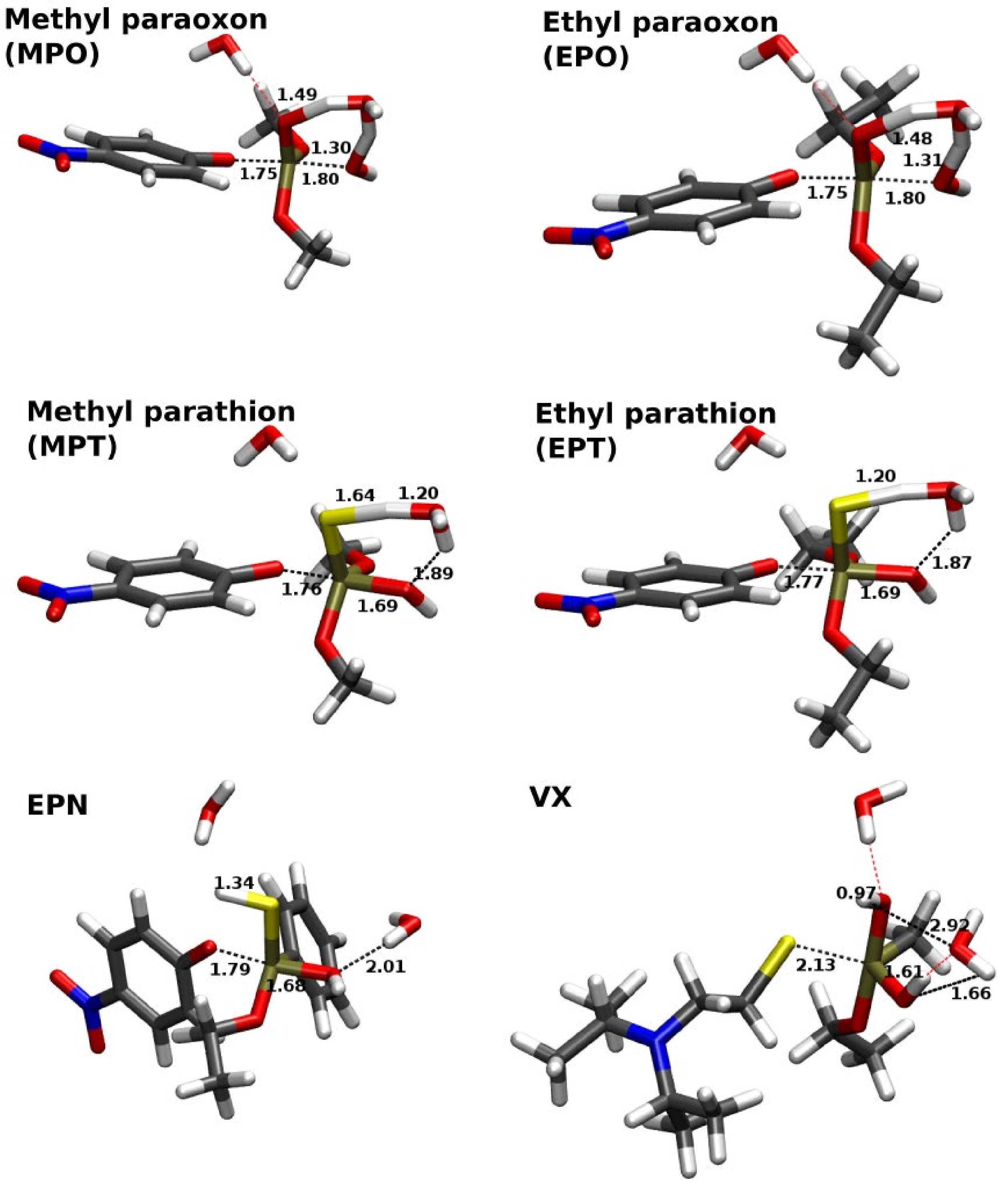

Fig. S15. Transition states TS1 for the neutral water hydrolysis of all six organophosphate substrates. Distances (in Å) of atoms involved in the attacking, leaving bonds and the proton transfer are shown.

Hydrolysis of chemical warfare agent VX.

VX was selected as a representative of chemical warfare agents. The hydrolysis of VX was studied using same methodology as the organophosphate pesticides. Three explicit water molecules were included with the VX molecule. The system was described using PBEh-3c QM method. Single-point energies were calculated using PBE0. Unlike the organophosphate pesticides, the VX agent does not follow the two-step reaction mechanism. There is no (meta)stable intermediate state during the hydrolysis. Instead, following the attack of the water molecule, the phosphoester bond breaks and the VX decomposes directly into the product state. The single transition state, equivalent to the TS1 state of the other organophosphates, differs from the rest as well. The breaking phosphoester bond is formed between phosphor and leaving sulphur instead of oxygen. The bond is much longer than the phosphoester bond in the other organophosphates. The different geometry of VX also means that the attacking water molecule approaches from a different angle than strictly opposite the phosphoester bond. The potential reaction energy and reaction barrier of VX hydrolysis is shown in Table S4 and Fig. S16. We see that the reaction barrier is lower than any of the other organophosphates studied. This is consistent with the lower stability of the VX agent.

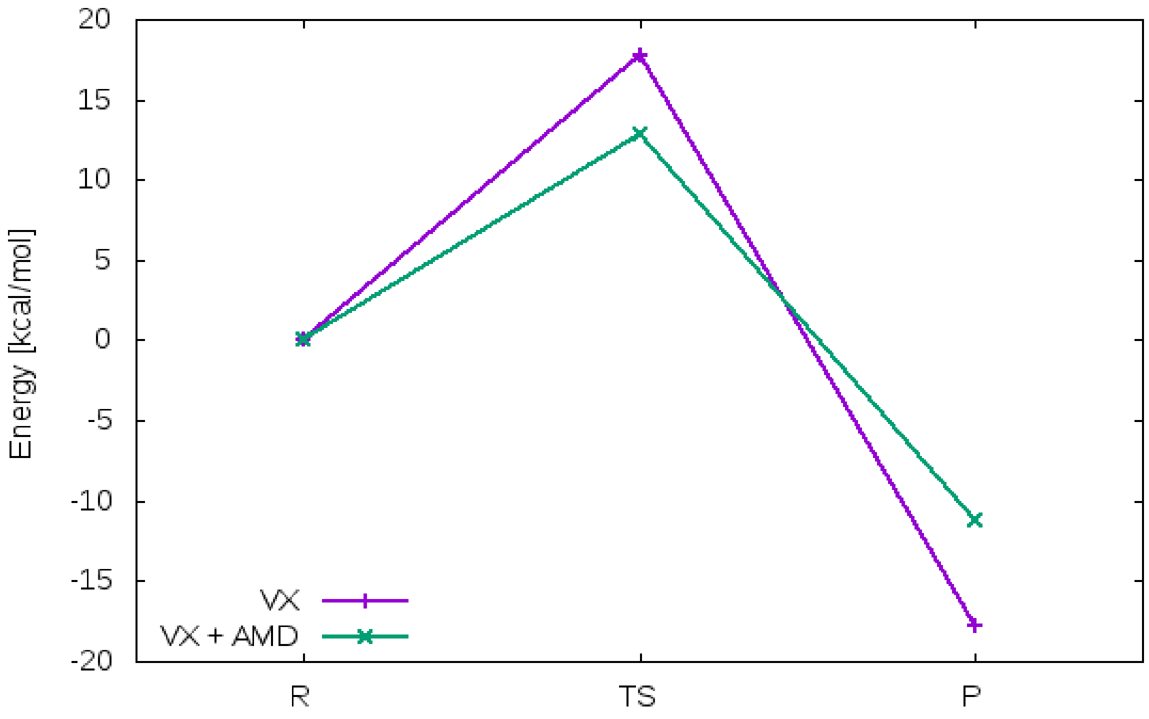

Fig.S16. Potential energies of VX hydrolysis relative to the respective reactant state (R) in kcal/mol calculated at the PBE0 level.

Degradation of organophosphates in the presence of the amidoxime-functionalized reactive cellulose

In order to investigate the degradation of organophosphates catalyzed by amidoxime-functionalized cellulose, a simplified model was considered. The model consisted of a single glucose molecule functionalized with amidoxime at the C6 position. Only parathion methyl and VX agent were considered in this part of the study. Classical molecular dynamics simulation of this functionalized glucose with a single parathion methyl molecule was performed to obtain orientation of these two molecules. The parathion methyl molecule was described by a GAFF force-field with RESP charges [11]. The glucose was described by GLYCAM force-field [12] and the amidoxime was described again by the GAFF force-field with RESP charges. TIP3P model was used for the explicit water molecules [11] MD simulations were performed using AMBER simulation package [12]. Suitable low energy orientation with a low distance between amidoxime group and phosphorus atom of parathion methyl was selected from 200 ns trajectory. Two explicit water molecules were included with the modified glucose and MPT. Long range solvent effects were included using COSMO implicit solvation method. Initial structures (R, TS1, INT, TS2, P) were constructed based on the corresponding structures from the hydrolysis in water. The structures were optimized using PBEh-3c and single-point energies were calculated using PBE0.

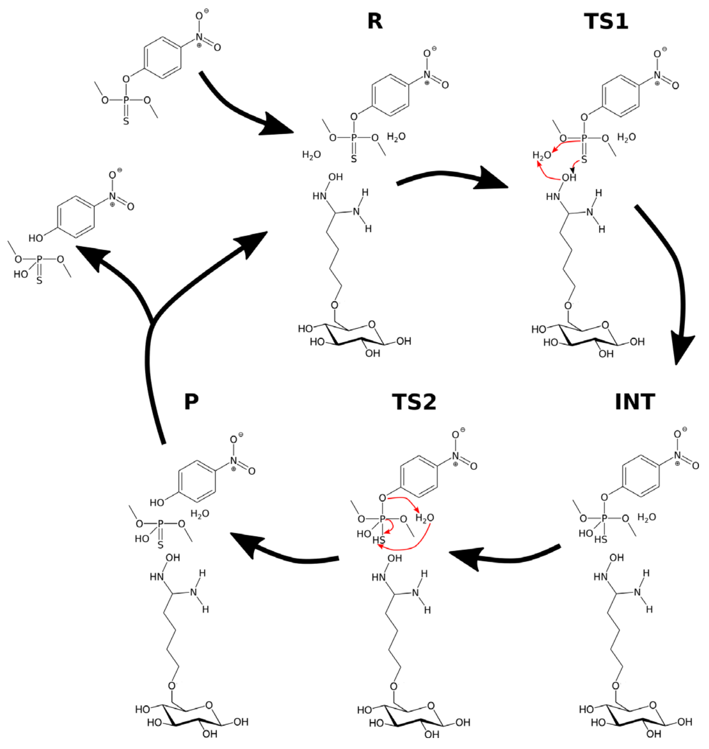

Fig. S17. Scheme of hydrolysis of parathion methyl catalysed by the amidoxime-functionalized cellulose.

Table S4. *Potential energies relative to the reactant state (R) in kcal/mol calculated at the PBE0 level*

	parathion methyl	parathion methyl + AMD
**TS1**	**31.40**	**11.37**
INT	20.26	8.90
TS2	26.57	8.93
P	-16.79	-5.02

The relative potential reaction energy, intermediate state energy and energy barriers associated with the two transition states are shown in Table S3 and Fig. S18. From the result, we see that the barrier of the first and rate-limiting step (TS1) was reduced by 20 kcal/mol to 11.37 kcal/mol (36% of the original barrier). This represents a significant reduction in reaction barrier. The energy values of the intermediate (INT) state and the second reaction barrier are also reduced compared to the water hydrolysis. The difference in the energy between INT state and the second barrier is smaller than the expected accuracy of the computational method. Indicating that the intermediate state may no longer be relevant in the oxime-catalysed hydrolysis. The transition state TS1 of the amidoxime-catalysed hydrolysis of MPT is shown in Fig. S18. The transition state is similar to the transition state in the water hydrolysis, but with shorter distances for both the leaving oxygen and the attacking oxygen.

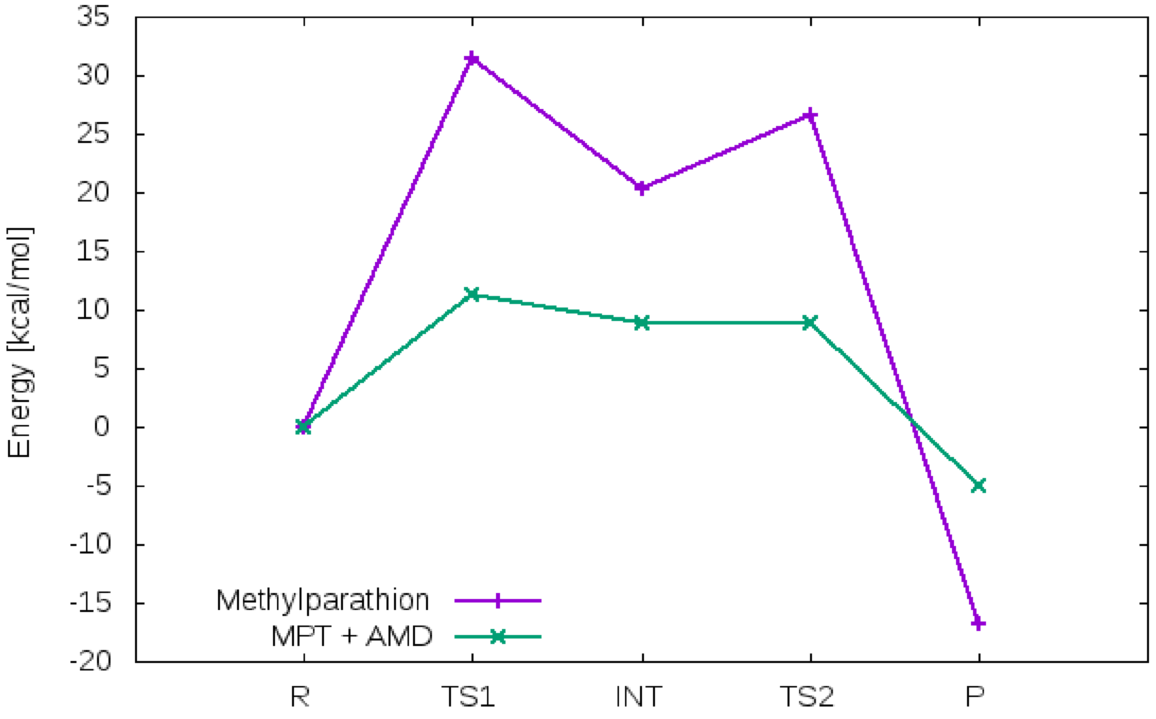

Fig. S18. Potential energies of the parathion methyl hydrolysis relative to the respective reactant state (R) in kcal/mol calculated at the PBE0 level.

Hydrolysis of VX using oxime functionalized cellulose.

The orientation of parathion methyl and modified glucose was used to construct the complex of VX and the modified glucose. Two explicit water molecules were included with the modified glucose and VX. Long range solvent effects were included using COSMO implicit solvation method. Initial structures (R, TS, P) were constructed based on the corresponding structures from the hydrolysis in water. They were then optimized using PBEh-3c and single-point energies were calculated using PBE0, same as previous calculations. As was mentioned previously, the hydrolysis of VX does not follow the two-step mechanism of the other organophosphates. Same was assumed for the amidoxime-catalysed hydrolysis of VX. The relative reaction energy and reaction barrier are shown in Table S4 and Fig. S18. The reaction barrier of the amidoxime-catalysed hydrolysis compared to water hydrolysis is reduced by almost 5 kcal/mol to 12.88 kcal/mol (72% of the original barrier). This reduction in the barrier height is much less pronounced than in the case of parathion methyl. In fact, the barrier of amidoxime-catalysed hydrolysis of VX is higher than the barrier of the amidoxime-catalysed hydrolysis of parathion methyl. Albeit only by about 1 kcal/mol, which is at the accuracy limit of the methodology. The transition state of the amidoxime-catalysed hydrolysis of VX is shown in Fig. S19. The transition state is similar to the transition state of water hydrolysis of VX and we do not see any shorting of leaving or attacking bonds as observed in the TS1 of amidoxime-catalysed hydrolysis of parathion methyl.

Table S4. *Potential energies of VX hydrolysis relative to the respective reactant state (R) in kcal/mol calculated at the PBE0 level*

	VX	VX + AMD
**TS**	**17.79**	**12.88**
P	-17.80	-11.19

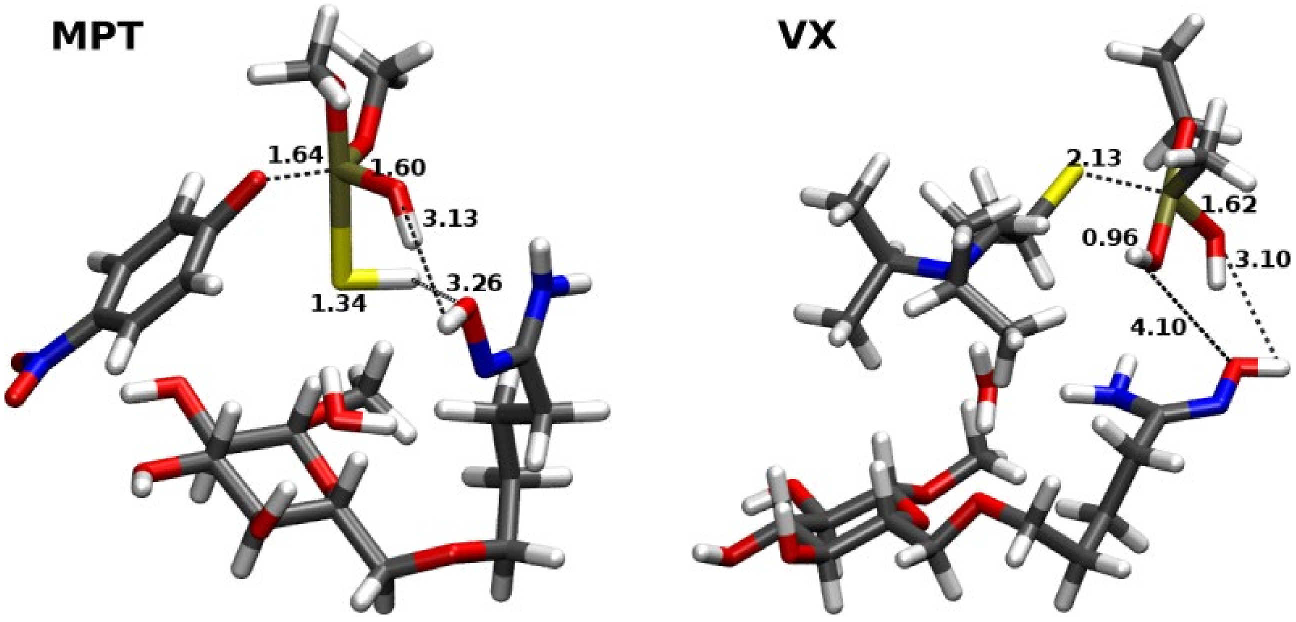

Fig. S19. Transition states TS1 for the amidoxime-catalysed hydrolysis of parathion methyl and VX. Distances (in Å) of atoms involved in the attacking, leaving bonds and the proton transfer are shown.

Table S5. *Partial atomic charges on selected atoms in the transition state.*

Atom	parathion methyl	parathion methyl + AMD	VX	VX + AMD
P	0.8111	0.8232	0.6876	0.7382
O_{attack}	-0.6636	-0.4331	-0.4499	-0.5528
O/S_{leave}	-0.3048	-0.2762	-0.1995	-0.1930
O_{AMD}	-	-0.4159	-	-0.4208
N_{AMD}	-	-0.4740	-	-0.4591

References:

[1] Chagas MA, Pereira ES, Da Silva JCS, Rocha WR. Theoretical investigation of the neutral hydrolysis of diethyl 4-nitrophenyl phosphate (paraoxon) in aqueous solution. J Mol Model. 2018;24(9):259.

[2] Grimme S, Brandenburg JG, Bannwarth C, Hansen A. Consistent structures and interactions by density functional theory with small atomic orbital basis sets. J Chem Phys. 2015;143(5):054107.

[3] Adamo C, Barone V. Toward reliable density functional methods without adjustable parameters: The PBE0 model. J Chem Phys. 1999;110(13):6158–6170.

[4] Klamt A. The COSMO and COSMO-RS solvation models. Wiley Interdiscip Rev Comput Mol Sci. 2011;1(5):699–709.

[5] Neese F. The ORCA program system. Wiley Interdiscip Rev Comput Mol Sci. 2012;2(1):73–78.

[6] Neese F. Software update: the ORCA program system, version 4.0. Wiley Interdiscip Rev Comput Mol Sci. 2018;8(1):e1327.

[7] Wang J, Wolf RM, Caldwell JW, Kollman PA, Case DA. Development and testing of a general amber force field. J Comput Chem. 2004 Jul 15;25(9):1157–74.

[8] Kirschner KN, Yongye AB, Tschampel SM, González-Outeiriño J, Daniels CR, Foley BL, et al. GLYCAM06: A generalizable biomolecular force field. Carbohydrates. J Comput Chem. 2008 Mar;29(4):622–55.

[9] Kamiya M., Mitsuhashi S, Makino M. Catalytic properties of cyclodextrins on the hydrolysis of parathion and paraoxon in aquatic medium containing humic acids. Chemosphere 1992; 25(12): 1783-1796.

[10] Sato R, Kubo H, The role of hydrolysis of some organophosphates. Chemical studies on organophosphorus pesticides. Botyu-Kagaku 1959; 24: 89-93.

[11] Jorgensen WL, Chandrasekhar J, Madura JD, Impey RW, Klein ML. Comparison of simple potential functions for simulating liquid water. J Chem Phys. 1983;79(2):926.

[12] Case DA, Babin V, Berryman JT, Betz RM, Cai Q, Cerutti DS, et al. Amber 14. University of California, San Francisco; 2014.
